# Validating Species Distribution Models With Standardized Surveys for Ixodid Ticks in Mainland Florida

**DOI:** 10.1093/jme/tjaa282

**Published:** 2021-01-02

**Authors:** Gregory E Glass, Claudia Ganser, William H Kessler

**Affiliations:** Department of Geography and Emerging Pathogens Institute, University of Florida, Gainesville, FL

**Keywords:** tick, surveillance, tickborne disease, external validity, species distribution model

## Abstract

Tick-borne pathogens are of growing concern. The U.S. Centers for Disease Control and Prevention (CDC) developed guidelines standardizing surveys of tick vectors to better monitor the changes in their occurrences. Unbiased surveillance data, from standardized surveys, are presumed critical to generate valid species distribution models (SDMs). We tested previously generated SDMs from standardized protocols for three medically important ticks [*Amblyomma americanum* (Linnaeus, Ixodida, Ixodidae), *Ixodes scapularis* (Say, Ixodida, Ixodidae), and *Dermacentor variabilis* (Say, Ixodida, Ixodidae)]. These previous models ruled out a quarter to half of the state as having these species, with consensus occurrence in about a quarter of the state. New surveys performed throughout 2019 on 250 transects at 43 sites indicated the rule-out functions were 100% accurate for *I. scapularis* and *D. variabilis* and 91.9% for *A. americanum*. As SDM concordance increased, the proportion of transects yielding ticks increased. Independent surveys of SDMs provide external validation—an aspect missing from many SDM studies.

Recent attention has focused on hard-bodied (ixodid) ticks and their pathogens (tickborne diseases [TBD]) as a growing public health concern. The emergence has been both in the expansion of geographic ranges and in the increased incidence of the associated diseases (Eisen et al. 2017, Beard et al. 2019, Eisen 2020). The U.S. Centers for Disease Control and Prevention (CDC) has developed guidelines for standardized surveys of these ticks to better monitor the extent and changes in their occurrences (CDC 2018, 2020). The standards ensure both that higher quality surveillance is performed and that gaps in knowledge are filled. Standardized survey protocols also reduce biases in the collected information—reducing analytical errors and erroneous interpretations (CDC 2018, Eisen 2020). These surveys generate local snapshots of occurrence or abundance. For example, the new recommendations propose sampling at least 750 m^2^ of transects for questing ticks using one of a few survey methods (CDC 2018, 2020). The data generate detailed information on species, life stage, and density. A nationwide, coordinated tick surveillance effort can only be achieved via local implementation of standardized surveillance methods.

Extrapolating local surveillance results to state, regional, or national levels require extended, explicit analyses. During the past several decades, ecologists and biogeographers have developed algorithms to create species distribution models (SDMs). The algorithms associate point data of the presence/absence/abundance of species at individual locations with more broadly sampled environmental conditions (Elith et al. 2006, Pearson et al. 2006, Bahn and McGill 2013, Shabani et al. 2016, Hao et al. 2019). SDMs have been applied to several medically important tick species in the United States both regionally and nationally (Glass et al. 1994, Das et al. 2002, Springer et al. 2015, Eisen et al. 2016).

An early recognized issue of SDMs was that biased local survey data influenced predictions with significant consequences (Reddy and Davalos 2003, Vaughan and Ormerod 2005, Newbold et al. 2010, Fourcade et al. 2014, Fithian et al. 2015). For example, in their original context, SDMs were used to identify critical environments of endangered species but modelers found that biased data incorrectly identified areas needed for protection (Reddy and Davalos 2003, Newbold et al. 2010, Bahn and McGill 2013). The analogous situation with TBD is that distribution maps generated from biased sampling may misidentify regions at risk for disease (errors of commission)—and more relevantly, omit areas where risk is elevated (errors of omission). Standardized survey protocols could help resolve this challenge.

We previously applied the CDC standardized survey strategy in mainland Florida (Glass et al. 2019) to estimate the geographic distributions of adult *Amblyomma americanum* (Linnaeus), *Ixodes scapularis* (Say), and *Dermacentor variabilis* (Say) by generating an ensemble SDM for each species (Kessler et al. 2019). Here, we evaluate the models, using independent validation surveys of questing adult tick presence/absence (Newbold et al. 2010, Bahn and McGill 2013). Overall, the ensembles performed well, ruling out large portions of the state as containing questing adults. Perhaps more importantly, during validation, few locations yielded questing ticks where the SDMs predicted them to be absent.

## Materials and Methods

The initial field surveys (CDC 2018, 2020) for three species of ixodid ticks—*A. americanum*, *I. scapularis*, and *D. variabilis*—are described elsewhere (Glass et al. 2019, Kessler et al. 2019). Briefly, 560 transects were located at 41 sites within mainland Florida between late 2015 and late 2018. These transects averaged 153 m (±2 m; SE) in length. The same transects were surveyed repeatedly throughout the seasons for 3 yr, applying CDC surveillance protocols. Collected ticks (primarily adults and nymphs) were removed from the flags and stored at −80°C until identified with a microscope (Glass et al. 2019). To evaluate whether variation in transect length due to sampling/GPS errors affected the likelihood of tick detection, during validation, lengths of transects were associated with whether at least one tick was collected or not (binary outcome) and tested as a simple logistic regression analysis.

In addition to the ticks, collection date, transect identifier, and longitude and latitude for the transect were obtained. The data were imported into a relational database and linked with selected environmental data of climate, vegetation condition, elevation, slope, aspect, soil conditions, land use/land cover, and geomorphology (Kessler et al. 2019).

From these data, we previously reported the results of an ensemble of five SDMs generated in R ver 3.6.3 (Kessler et al. 2019). The component models were as follows: a general linear model (logistic regression with a logit link function), multivariate adaptive regression splines, boosted regression trees, random forests, and MaxEnt models. The models generated continuous estimates for the probabilities of tick occurrence for each species, and the ensemble results were dichotomized using a probability threshold that yielded equal specificity and sensitivity (Jimenez-Valverde and Lobo 2007). The five dichotomized models were overlain, so each pixel in mainland Florida scaled between 0 and 5 (no model predicted species presence—all models predicted species presence).

New, validation surveys were performed from January to December 2019. The protocol repeated the original field survey methods (Glass et al. 2019). During validation, 43 sites were chosen. Twenty-five new sites were identified, and 18 sites also were surveyed during the initial studies. The 18 previous sites were retained to determine whether deviations during validation were due to surveys in different years (Newbold et al. 2010). If the new validation sites were poorly predicted by the ensemble, while the repeated sites were consistent with the 2015–2018 surveys, we interpreted the discrepancy as poor specification by the models rather than differences in tick abundances across years.

Standard epidemiologic descriptors were calculated to evaluate the ensemble (Kelsey et al. 1996). Negative and positive predictive values (NPV and PPV, respectively) as well as sensitivity and specificity were calculated from 2 × 2 tables where each tick species was ‘positive’ if it was found on a transect at any time during the validation or ‘negative’ if it was never sampled during the validation year. The ensemble predicted ‘present’ if any of the five models predicted occurrence for the tick species at the transect and ‘absent’ if none of the five models predicted occurrence at the transect. These associations were further examined by identifying the proportion of transects yielding each species of tick (number of transects ‘positive’/total number of transects) and compared with agreement among the SDMs for each transect. SDM model agreement was grouped into 0, 1–2, 3–4, and 5 models predicted presence. Transects where a tick species was found but 0 models predicted presence were considered errors of omission for the ensemble. Distances to the nearest pixels with a predicted occurrence by SDMs were calculated in GIS. Photographs of the ‘omission error’ transects collected during surveys were examined to identify aspects of the local physical environment not captured in the environmental databases. Transects where ticks were predicted to occur by at least one (and up to five) models but were identified as ‘absent’ were considered as errors of commission.

## Results

**Fig. 1.**
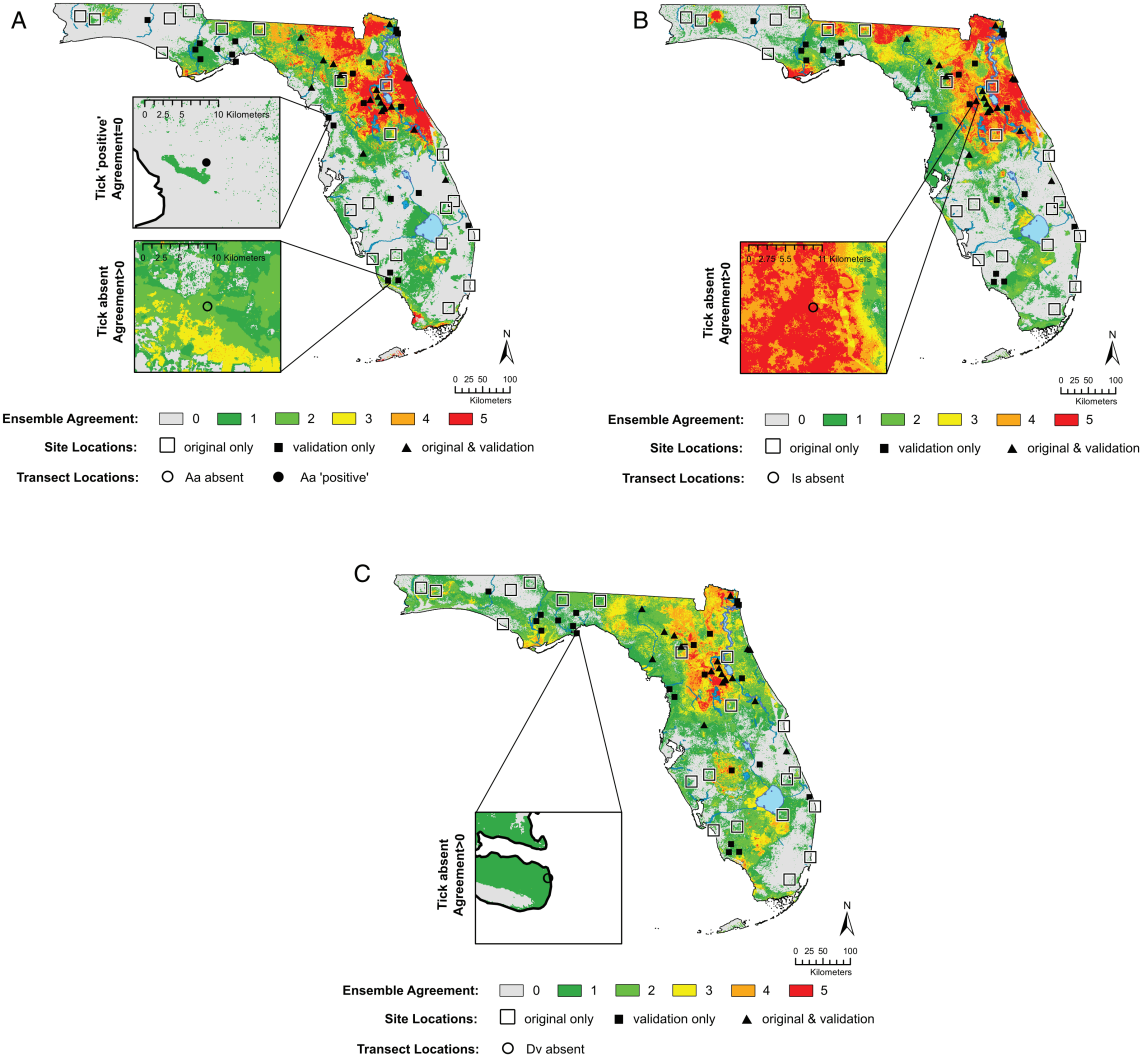
(A) Ensemble model prediction for *Amblyomma americanum* and sampling locations for 41 sites in the original study (open boxes and filled triangles) used to generate ensemble species distribution models (SDMs) (Kessler et al. 2019). Color scheme for model agreement is from Kessler et al. (2019), with gray = no models predicted occurrence through green (one or two models), yellow (three models agree), orange (four models agree), or red (all models predict occurrence). Filled triangles were surveyed during the original survey and during validation. Filled boxes indicate new validation survey sites. Insets in (A) show an example of a transect at the site with an omission error (the transect was found to be ‘tick positive’ for *A. americanum*, but no ensemble model predicted occurrence [Agreement = 0]), and an example of commission error (open circle; transect where questing *A. americanum* were not found but ensemble model predicted occurrence ‘Agreement > 0’). (B) Ensemble model prediction for *Ixodes scapularis* with same original and validation sites. Color scheme is as in (A). Inset only demonstrates an example of a site with a ‘commission error’ transect, no errors of omission were found for *I. scapularis*. (C) Ensemble model prediction for *Dermacentor variabilis*. Color scheme is as in (A). Inset only demonstrates an example of a site with a commission error transect, no errors of omission were found for *D. variabilis*.

Previously generated ensemble models (Kessler et al. 2019) predicted the three species occupied substantially reduced extents of the mainland, depending on the number of SDMs used to identify a ‘suitable’ region (Table 1; Fig. 1). Generally, the largest contiguous regions, with the greatest consensus, were in the north central part of the state. Fewer models predicted species occurrences toward the south or into the panhandle (northwest). Notably, some of the SDMs predicted regions of suitable environments extending from Lake Okeechobee and along the southern edge of the mainland, for all species.

**Table 1.** The proportion of mainland Florida identified as suitable for three tick species by none of the SDMs, one or two models, three or four models, or all five SDMs

Concordance	*Amblyomma americanum*		*Ixodes scapularis*		*Dermacentor variabilis*	
	% area (km^2^)	% trans. (no. of trans.)	% area (km^2^)	% trans. (no. of trans.)	% area (km^2^)	% trans. (no. of trans.)
None	46.6 (68,388)	8.1 (75)	39.5 (57,968)	0.0 (51)	26.0 (38,156)	0.0 (35)
1–2	30.8 (45,200)	19.7 (81)	33.0 (48,428)	8.2 (110)	50.9 (74,698)	1.5 (135)
3–4	13.0 (19,078)	41.7 (36)	18.0 (26,416)	10.2 (39)	21.2 (31,112)	7.4 (54)
5	9.6 (14,088)	50.0 (58)	9.4 (13,795)	56.0 (50)	1.9 (2,788)	19.2 (26)

For example, 9.4% of the state was deemed suitable for *I. scapularis* by all five models and this covered 13,795 km^2^. Fifty-six percent of the 50 transects in this region yielded *I. scapularis*. Large, but varying, portions of the state were predicted unsuitable for each species (concordance = none). The total land area of mainland Florida was estimated as 146,754.6 km^2^ (Kessler et al. 2019). SDM, species distribution model; trans., transect.

Validation survey sites were widely distributed throughout the mainland, including regions where ensemble models predicted species would be absent (Fig. 1). In 2019, the 43 validation sites included 250 transects that were surveyed 1450 times on an approximately bimonthly schedule, although this varied with local conditions. The numbers of transects ranged from 2 to 17 at each site (median = 4 transects) based on the size of the site and existing land cover classes (Glass et al. 2019). Eighteen sites and their 105 transects had been sampled during ensemble model development (2015–2018) and were resampled for validation. Twenty-five sites, with 145 transects were surveyed for the first time during 2019 (Fig. 1; Supp Table 1 [online only]).

The average length of transects was 156 m ± 2.5 m (*x* ± SE), which was not significantly different from the 2015 to 2018 transect lengths. The validation transects where ticks were collected did not differ significantly in length from validation transects where ticks were not collected (odds ratio = 0.99; 95% confidence interval = 0.98–1.01).

Transects where ticks were collected in validation surveys were consistent with those from 2015 to 2018 (Supp Table 1 [online only]). Of the 18 resampled sites, 64.8% (35/54, where the denominator is 18 sites × 3 species) agreed between model development (2015–2018) and validation (tick species either present or absent). Among discordant sites, 29.6% (16/54) yielded tick species during model development but not during validation. These sites were where a species was rarely observed during 2015–2018 (only one or two transects scored ‘present’; Supp Table 1 [online only]). For example, *A. americanum* was found one time on a single transect at Colt Creek during 2015–2018 (1/13 transects during 158 surveys) and on no transects during 2019 (Supp Table 1 [online only]). *Dermacentor variabilis*, which were sporadically captured throughout the studies generated most of the discordant survey results (7/18 sites). Rarely, a species was first observed at a site during validation when it had not been detected during model development. There were three sites (one for each species) where a species was first collected in 2019 and they only were found on single transects. These three transects fell within predicted occurrence regions of the ensemble models.

Overall, the proportion of transects yielding adult *D. variabilis*, *I. scapularis*, or *A. americanum* ranged from 4.0 to 26.4% (Table 2). Lone star ticks were most frequently sampled (60/250 transects), whereas black-legged ticks somewhat less commonly (41/250 transects) and dog ticks were least frequently documented (10/250 transects; Table 2). Despite the wide variation in the transects yielding different species, all species were geographically widespread (Supp Table 1 [online only]).

**Table 2.** Comparison of validation surveys (columns; transects positive/negative) with SDM predictions at the transect (rows; model indicates present/absent) and summary measures of evaluation (±95% CI)

Model	*Amblyomma americanum*			*Ixodes scapularis*			*Dermacentor variabilis*		
	Positive	Negative	Total	Positive	Negative	Total	Positive	Negative	Total
Present	60	116	176	41	158	199	10	204	214
Absent	6	68	74	0	51	51	0	36	36
Total	66	184	250	41	209	250	10	240	250
Measure									
Sensitivity	90.9 (81.3, 96.6)			100.0 (91.4, 100.0)			100.0 (69.2, 100.0)		
Specificity	37.0 (30.0, 44.4)			24.4 (18.7, 30.8)			15.0 (10.7, 20.2)		
PPV	34.1 (31.1, 37.2)			20.6 (19.4, 21.9)			4.7 (4.4, 4.9)		
NPV	91.9 (83.8, 96.1)			100.0			100.0		

CI, confidence interval; PPV, positive predictive value; NPV, negative predictive value; SDM, species distribution model.

When ensemble models (Kessler et al. 2019) were dichotomized; (no SDM predicted occurrence vs at least one SDM predicted occurrence on a transect), the NPV (ensemble models ruling out transects as yielding adult ticks), was consistently high (Table 2). NPV was 100.0% for *I. scapularis* and *D. variabilis.* The NPV for *A. americanum* was also high (91.9%), but adult lone star ticks were found on six transects where they were not predicted to occur. Similarly, the sensitivity was high, so that ticks were nearly exclusively found on transects predicted by the SDMs (Table 2).

**Fig. 2.**
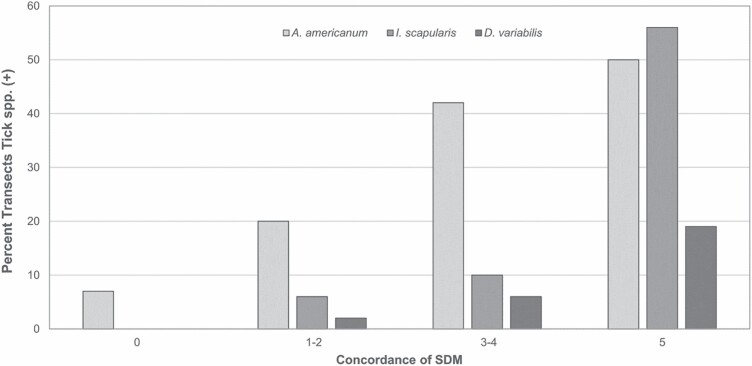
Proportion of validation transects yielding adult questing tick species as species distribution model (SDM) agreement increased. Transects with 0 concordance had all SDMs predict that tick species would be absent. Transects with five concordance had all SDMs predict that specific tick species would be present. Vertical axis is the percentage of transects in the concordance categories (Table 1) that yielded specific tick species.

In contrast, many transects that the SDMs predicted should yield ticks did not (commission errors) producing overall, low estimates of PPV and specificity (Table 2). This seems to have been driven predominantly by predictions from a minority of the SDMs. From 30.8 to 50.9% of the state was predicted to have adult questing ticks based on only one or two SDMs. The, overall, low predictive values improved as agreement among the SDMs increased, so that when the preponderance of SDMs (3, 4, or 5 models) predicted occurrence, transects yielding ticks were highest (Fig. 2). Concordance occurred in 23–27% of the mainland (Table 1) and presumably represented the highest likelihood of exposure to questing ticks (Kessler et al. 2019).

The 6/74 transects the models predicted would not have *A. americanum* were omission error transects (Table 2). These transects were near where SDMs predicted occurrence (Fig. 1A, inset for example). All six transects were within a half kilometer of suitable habitat and half were within 300 m (=3 pixels). Notably, these ‘omission error’ transects were on a specific landform in northern and western coastal Florida. These transects were atop abandoned commercial trams in swamps that were used to harvest cypress.

## Discussion

TBD surveillance protocols identify criteria that are intended to improve the quality of information about the risks, the timing, and locations of vectors and their pathogens (Eisen et al. 2017; CDC 2018, 2020; Beard et al. 2019; Eisen 2020). Standardized survey methods have a long history in epidemiology (Nelson et al. 2014) and should also improve TBD data. Methods using local surveys to estimate spatial patterns of potential TBD risk also have emerged in recent years (Springer et al. 2015). Early approaches at regional and national levels relying only on expert opinion provided poor spatial detail (Sonenshine 2018). SDMs, borrowing heavily from ecological and biogeographic approaches, formalized techniques that linked surveys with more extensive environmental data. Numerous strategies to create SDMs have been developed, differing in their underlying methods and assumptions, and producing similar but distinct outcomes (Elith et al. 2006, Pearson et al. 2006, Shabani et al. 2016, Hao et al. 2019). Borrowing a framework more commonly applied in meteorology, outputs from multiple SDMs have been combined into ‘ensemble’ models (Araújo and New 2007, Franklin 2010) and were more accurate than any single model (Araújo and New 2007, Hao et al. 2019).

It has been recognized for more than a decade that SDM algorithms are blind to biased data when they are used as to generate models (Reddy and Davalos 2003, Kramer-Schadt et al. 2013, El-Gabbas and Dormann 2018, Hao et al. 2019). Developers have warned that using biased location data generate misleading results (Elith et al. 2006, Bahn and McGill 2013, Kramer-Schadt et al. 2013, Fourcade et al. 2014, Fithian et al. 2015, Shabani et al. 2016, El-Gabbas and Dormann 2018). In Florida, validation surveys that used CDC guidelines gave results consistent with the SDMs, especially in ruling out large regions of the state. The 1-yr validation surveys rarely collected ticks where they were not observed previously. Validation surveys tended to find ticks at most sites where they had frequently been previously recorded, much as reported in other standardized surveys (Newbold et al. 2010, El-Gabbas and Dormann 2018).

The discordance of the validation results with ensemble models occurred primarily at previously sampled sites that yielded ticks only sporadically during the model building phase, so the SDMs were ‘positive’ but did not produce ticks during validation. This may suggest either imperfect detection because of flagging methods and/or high rates of unoccupied, suitable habitat patches due to local extinction in the absence of recolonization (occupancy effects) that may affect the accuracy of the ensemble classification. Regardless, the 3-yr model building effort identified many, but not all specific locales where the ticks were recovered (Supp Table 1 [online only]). By sampling based on land cover and climatic zones of the state (Glass et al. 2019), the original 41 sites generated ensemble models that effectively excluded large regions of questing adult tick activity (Fig. 1; Table 1).

The ensemble models were less successful in identifying transects within the predicted tick region that yielded ticks than the models were in excluding areas that did not have ticks (Fig. 2). Much of the low specificity and PPV was driven by the 81–135 transects (Table 1) conducted in areas where only 1–2 SDMs predicted tick species occurrence. Transects in these areas only rarely, yielded the target species.

The ensemble models rarely generated errors of omission—an important characteristic of a screening tool. The only six transects that ‘failed’ were for *A. americanum* and were located near (<0.5 km) suitable locations. They represented a single human-generated landform (tram trails) in otherwise water-saturated conditions. Trams remain as recreational paths and provide routes for medium—large terrestrial mammals in the region. The widths of the tram beds are narrower than the nominal spatial resolution (100 m) of the environmental data (Kessler et al. 2019). These physically narrow habitats (<10 m in width) were not recorded in the landcover database with its original resolution (Kessler et al. 2019)—a testament to the impact of discordance between database spatial resolution and ecological suitability of vectors. This specific human-generated environment is now rarely created, and we anticipate little physical expansion of this unique land use pattern.

Commission errors (predicting tick occurrence without being detected) were more common (Table 2). Questing ticks are recognized to be ‘spotty’ or aggregated in many regions. Current analytical approaches tend to smooth these effects by averaging sampling results from multiple transects at individual sites. However, earlier studies (Falco and Fish 1989, Telford et al. 1992, Duffy et al. 1994) found that even in hyperendemic areas for *I. scapularis*, transects without nymphs ranged between 12 and 67%, indicating highly local clustering of questing ticks. These studies varied in their survey methods, often using time rather than transect lengths, so that the current, standardized survey protocols may help reduce much of this variability.

Omission and commission error rates can also be influenced by the method used to select the threshold for presence–absence from the continuous surfaces of the SDMs (Jimenez-Valverde and Lobo 2007). Kessler et al. (2019) applied the frequently used ‘equal sensitivity and specificity’ in their final presentation, even though there are various other threshold criteria (Fielding and Bell 1997, Hahn et al. 2017). Kessler et al. (2019) evaluated seven thresholds (not shown). Five of those generated comparable thresholds to equal sensitivity and specificity, while using the default criteria of ‘0.5’ produced the only obvious outlying results. Regions with consensus agreement among the SDMs was a good indicator of increased proportion of transects with tick captures—reaching more than 50% when all SDMs predicted presence (Fig. 2). Sites where three or more of the models identified ticks as present yielded ticks on substantially more transects than transects located where only 1–2 models predicted occurrence.

Despite the overall success applying standardized formats to ensemble SDMs (Table 2), there are challenges. The format presumes a prospective study design. Under some circumstances, such as case investigations, that design may not be feasible or necessary (Savage et al. 2013, 2017; Jackson et al. 2019). Similarly, determining if an invasive species, such as *Haemaphysalis longicornis* (Neumann, Ixodida, Ixodidae), occurs locally may not require a surveillance framework, though it would be beneficial, if the goal is to later extrapolate to unsampled regions.

Standardized surveys also suppose financial, staffing, and equipment resources to execute the work are available. It is often true that resources for field surveys usually are sacrificed even when laboratory resources are allocated to test samples and interpret results. Consequently, many vector-borne SDM studies rely on serendipitously acquired data, often gathered haphazardly (not to be confused with randomly) that are repurposed to the task at hand. In principle, such ‘best available data (BAD)’ can initiate risk assessments, but they should be used cautiously until validated. Until validation is performed, the quality of SDMs predictions from biased data remains uncertain (Vaughan and Ormerod 2005, Fourcade et al. 2014, Hao et al. 2019).

Methodologically, SDMs generated from BAD assess model quality by subsampling within the repurposed data set, generating hold-out samples that are compared with the model outcomes (Fielding and Bell 1997, Elith et al. 2006). The consistency of the identified environmental predictors and the behavior of the model variants are used to characterize the robustness of the results. Empirical studies show that these strategies produce maps that are overwhelmingly optimistic compared with those generated from new, and independent, samples (Newbold et al. 2010, Bahn and McGill 2013). In epidemiology, this type of analysis evaluates internal validity that gauges the likelihood that specific, environmental predictors are associated with changes in the observed data set (tick occurrence or abundance).

However, external validity considers whether the results can be generalized to different times and places (Kelsey et al. 1996), instead of the specific database, and may be more relevant (Vaughan and Ormerod 2005). External validity is usually the provenance of the study design prior to data collection and analysis rather than the analytical methods that are applied. SDMs from convenience samples produce output (maps) that may have repeatable results (internal validity), but they may not be generalizable to other locations or times, including internal geographic regions (external validity) if they are biased.

Validation should be extended further to explore the standardized survey strategy (CDC 2018, 2020). Using the ensembles to predict species presence by examining other life stages or other survey methods would increase confidence in the standardized survey data. Although we were limited to a single year in advance, historical data also could be used to hindcast vector distributions. Various factors, such as historical human population distribution and land use change, would need to be incorporated rather than assuming the local conditions are unchanged.

Even biased convenience sampling, such as museum specimens or citizen science reports, could help validate standardized survey approaches. If SDMs from the standardized surveys are accurate, they should predict occurrence of convenience samples, regardless of their biases. However, we anticipate that this relationship will be asymmetric. We expect that the standardized survey SDMs will predict convenience sample outcomes more accurately than convenience sample generated SDMs predict the validation data.

## Supplementary Data

Supplementary data are available at *Journal of Medical Entomology* online.

Supplementary Table 1. Sites for tick surveys, the numbers of transects surveyed per site and the numbers of transects yielding adult ticks, by species during validation (2019) and original surveys (2015–2018). Original sites were sampled during surveys from 2015 to 2018 and during validation (2019). New sites were only surveyed during 2019.

tjaa282_suppl_Supplememtary_Materials
