# Supplementary material for: Validating Species Distribution Models With Standardized Surveys for Ixodid Ticks in Mainland Florida
Source: J Med Entomol. 2021 Jan 2;58(3):1345–51. doi: 10.1093/jme/tjaa282 (PMC8122235; doi:10.1093/jme/tjaa282)
Supplement: tjaa282_suppl_Supplememtary_Materials [file tjaa282_suppl_supplememtary_materials.docx]

**SUPPLEMENTARY MATERIAL TABLE S1.**

**Table 1**. Sites for tick surveys, the numbers of transects surveyed per site and the numbers of transects yielding adult ticks, by species during validation (2019) and original surveys (2015-2018). Original sites were sampled during surveys from 2015-2018 and during validation (2019). New sites were only surveyed during 2019.

| **Site Name** | **Long.^a^** | **Lat.**  **^b^** | **# Trans. ^c^** | **Validation Survey (2019)** | | |  | **Original Survey (2015-2018)** | | |
| --- | --- | --- | --- | --- | --- | --- | --- | --- | --- | --- |
|  |  |  |  | Aa^d^ | Is^e^ | Dv^f^ |  | Aa | Is | Dv |
| **ORIGINAL SITES** |  |  |  |  |  |  |  |  |  |  |
| Astor | -81.5 | 29.1 | 2 | 2 | 2 | 2 |  | 2 | 2 | 1 |
| Big Talbot | -81.4 | 30.5 | 17 | 1 | 15 | 0 |  | 15 | 17 | 1 |
| Colt Creek | -82.0 | 28.3 | 13 | 0 | 0 | 0 |  | 1 | 0 | 1 |
| Farles Lake | -81.7 | 29.1 | 4 | 2 | 0 | 1 |  | 4 | 3 | 4 |
| Faver Dyke | -81.3 | 29.7 | 4 | 4 | 0 | 0 |  | 4 | 2 | 0 |
| Fore Lake | -81.9 | 29.3 | 4 | 4 | 3 | 1 |  | 4 | 4 | 3 |
| Ichetucknee | -82.8 | 30.0 | 10 | 5 | 0 | 0 |  | 6 | 0 | 0 |
| Juniper Prairie | -81.7 | 29.2 | 2 | 0 | 0 | 0 |  | 1 | 0 | 0 |
| Lake Kerr | -81.7 | 29.3 | 2 | 1 | 0 | 0 |  | 0 | 0 | 0 |
| Oleno | -82.6 | 29.9 | 8 | 5 | 2 | 0 |  | 6 | 3 | 1 |
| Pinecastle | -81.8 | 29.1 | 4 | 2 | 0 | 1 |  | 4 | 2 | 3 |
| Flagler Beach | -81.2 | 29.7 | 3 | 1 | 3 | 1 |  | 2 | 2 | 0 |
| Rodman | -81.8 | 29.4 | 4 | 0 | 0 | 0 |  | 1 | 0 | 1 |
| San Felasco | -82.5 | 29.7 | 4 | 1 | 0 | 0 |  | 3 | 2 | 0 |
| Sellers Lake | -81.7 | 29.1 | 4 | 1 | 1 | 1 |  | 2 | 0 | 1 |
| St. Sebastian | -80.6 | 27.8 | 10 | 0 | 0 | 0 |  | 0 | 0 | 0 |
| Suwanee | -83.2 | 30.4 | 6 | 2 | 0 | 0 |  | 3 | 1 | 1 |
| Yulee | -81.6 | 30.6 | 4 | 0 | 2 | 0 |  | 1 | 2 | 2 |
| **Subtotal** |  |  | 105 | 31 | 28 | 7 |  | 59 | 40 | 19 |
|  |  |  |  |  |  |  |  |  |  |  |
| **NEW SITES** |  |  |  |  |  |  |  |  |  |  |
| Amelia Island | -81.4 | 30.5 | 2 | 0 | 0 | 0 |  |  |  |  |
| Austin Cary | -82.2 | 29.7 | 8 | 3 | 0 | 1 |  |  |  |  |
| Bald Point | -84.3 | 29.9 | 6 | 0 | 1 | 0 |  |  |  |  |
| Blanding | -81.9 | 29.9 | 12 | 7 | 4 | 1 |  |  |  |  |
| Camel Lake | -85.0 | 30.3 | 2 | 0 | 0 | 0 |  |  |  |  |
| Collier | -81.6 | 26.0 | 6 | 0 | 0 | 0 |  |  |  |  |
| Crystal River | -82.7 | 28.9 | 10 | 7 | 2 | 1 |  |  |  |  |
| De Leon | -81.4 | 29.1 | 4 | 0 | 1 | 0 |  |  |  |  |
| Devil’s Millhopper | -82.4 | 29.7 | 4 | 3 | 0 | 0 |  |  |  |  |
| Dickinson | -80.1 | 27.0 | 6 | 0 | 0 | 0 |  |  |  |  |
| Fakahatchee | -81.4 | 26.0 | 6 | 0 | 0 | 0 |  |  |  |  |
| Geneva | -81.1 | 28.7 | 3 | 3 | 1 | 0 |  |  |  |  |
| Highlands Hammock | -81.5 | 27.5 | 8 | 0 | 0 | 0 |  |  |  |  |
| Homosassa | -82.6 | 28.8 | 5 | 0 | 0 | 0 |  |  |  |  |
| Kissimmee | -81.0 | 27.6 | 12 | 0 | 0 | 0 |  |  |  |  |
| Leon | -84.3 | 30.3 | 4 | 2 | 0 | 0 |  |  |  |  |
| Little Talbot | -81.4 | 30.5 | 4 | 0 | 3 | 0 |  |  |  |  |
| Manatee Springs | -83.0 | 29.5 | 4 | 4 | 0 | 0 |  |  |  |  |
| Picayune | -81.6 | 26.1 | 12 | 0 | 0 | 0 |  |  |  |  |
| Ponce | -85.9 | 30.7 | 6 | 0 | 0 | 0 |  |  |  |  |
| Porter Lake | -84.7 | 30.2 | 3 | 1 | 0 | 0 |  |  |  |  |
| Rte379 | -85.1 | 30.2 | 4 | 0 | 0 | 0 |  |  |  |  |
| Silver Springs | -82.0 | 29.2 | 8 | 4 | 0 | 0 |  |  |  |  |
| Sopchoppy | -84.4 | 30.1 | 2 | 1 | 0 | 0 |  |  |  |  |
| Sumatra | -85.0 | 30.0 | 4 | 0 | 1 | 0 |  |  |  |  |
| **Subtotal** |  |  | 145 | 35 | 13 | 3 |  |  |  |  |

^a^Long.: Longitude of sampling sites
^b^Lat.: Latitude of sampling sites
^c^Aa: *Amblyomma americanum*
^d^Is: *Ixodes scapularis*
^e^Dv: *Dermacentor variabilis*
